# Development of Radiographic Radiation Pneumonitis (RP) in Non-small Cell Lung Cancer Patients Treated With Stereotactic Body Radiation Therapy (SBRT) May Be Protective Against Further Disease Progression

**DOI:** 10.7759/cureus.25994

**Published:** 2022-06-16

**Authors:** Colten Wolf, Michael Wesolowski, Kyle Stang, Fiori Alite, Matthew Harkenrider

**Affiliations:** 1 Otolaryngology - Head and Neck Surgery, Loyola University Medical Center, Maywood, USA; 2 Biostatistics, Loyola University Chicago Stritch School of Medicine, Maywood, USA; 3 Radiation Oncology, Loyola University Medical Center, Maywood, USA; 4 Radiation Oncology, Geisinger Cancer Institute, Lewisberg, USA; 5 Radiation Oncology, Loyola University Chicago Stritch School of Medicine, Maywood, USA

**Keywords:** immune system, lung toxicity, pneumonitis, stereotactic, lung cancer

## Abstract

Objectives: Radiation pneumonitis (RP) is a local inflammatory response, and we hypothesize that RP serves as an immune stimulator and is a protective factor against disease progression.

Methods: We analyzed patients with early-stage non-small cell lung cancer (NSCLC) treated with stereotactic body radiation therapy (SBRT) at two institutions. Radiographic RP (RRP) was evaluated and maximal axial dimensions were measured at three-, six-, and twelve-month timepoints with surveillance CT. RRP was measured using radiographic markers such as ground-glass opacities and airspace consolidation. Disease recurrence was evaluated and categorized as local, regional, and distant.

Results: Seventy-seven unique patient records were randomly selected from the database, 72 patients (93.5%) had RRP and five patients (6.5%) did not. The median follow-up was 24.3 months (IQR: 12.0 - 41.9). Disease failure occurred in 28.6% of patients with 6.5% local only, 2.6% regional only, 7.8% distant only, and 11.7% with multiple recurrences. Patients with RRP demonstrated a lower rate of disease failure with 25.0% of those with RRP experiencing disease failure and 80% of those without RRP experiencing disease failure (p=0.02). Patients with RRP had a 71% reduced risk of disease recurrence, compared to patients with no RRP, after adjusting for maximum tumor dimension (HR 0.29, p = 0.05). Among patients with RRP, there was no significant difference in recurrence based on extent of RRP (maximal area of RRP on CT). RRP did not correlate with overall survival.

Discussion: Most patients who received SBRT had RRP, and this study suggests that it may be protective of cancer recurrence. These results are hypothesis-generating and will need to be validated in larger and independent datasets.

## Introduction

Lung cancer is the leading cause of cancer death in both men and women in North America [1]. Classically, surgical resection has been the first line of treatment for patients with early-stage non-small cell lung cancer (NSCLC), however this mode of treatment is not well tolerated in patients with other comorbid disorders such as emphysema or heart disease [2]. Surgical resection for NSCLC is not feasible on every patient, and conventional radiotherapy fails to have significant effect with a local control rate of only 40-60% [3-6]. 

Stereotactic body radiation therapy (SBRT) is a non-invasive treatment that uses highly accurate and focused beams or arcs to deliver high doses per fraction with optimal image guidance over a few number of treatments. Studies have shown SBRT to be superior to conventional radiation therapy for early-stage inoperable NSCLC with local control rates routinely ≥85% [2,7-11]. 

Patients with inoperable tumors are often able to undergo treatment with SBRT and maintain good functional status. Poor pulmonary function tests (PFTs) at baseline do not decrease survival after SBRT, showing that poor PFTs should not exclude patients from SBRT [12]. In addition, there appears to be no dose-effect relationship for changes in PFT after SBRT [13]. One of the primary concerning toxicities associated with thoracic irradiation, including lung SBRT, is symptomatic radiation pneumonitis (RP) which occurs in around 10% of patients [14-16]. RP occurs due to a local inflammatory response of the lung parenchyma as a subacute response to the local irradiation. 

Radiation is well known to induce tumor cell death and some studies have shown an important role of the immune system in the therapeutic effect of radiation [17]. This data suggests that the immune system plays an important role in killing tumor cells within the area of irradiation. This relationship between the immune system and radiation is likely to involve a systemic immune response and peripheral activation of CD8+ T cells [18]. There have been reports that demonstrate that irradiation increases lymphocyte extravasation from circulating blood and subsequent infiltration of the tumor [19]. Moreover, SBRT may mediate cell kill through ablation of tumor vasculature, alteration of the tumor microenvironment, antigen release, and a local inflammatory response. Since SBRT tends to create this inflammatory response and increase in circulating CD8+ T cells, we hypothesize that lung cancer patients receiving SBRT who have a robust immune response, seen through the development of radiographic RP (RRP) on imaging, have improved disease control as a result of this enhancement to the immune system. The purpose of this study is to determine if presence of RRP decreases the risk of disease progression in a population of early-stage NSCLC patients treated with SBRT. 

## Materials and methods

We randomly selected patients from our database with early-stage NSCLC treated with SBRT. This patient cohort was from Loyola University Medical Center and Edward Hines Jr. VA Hospital with approval from the institutional review boards (IRB number LU205020). Inclusion criteria consisted of patients with NSCLC and were treated with primary SBRT. Patients were either deemed medically inoperable by a multi-disciplinary tumor board or declined surgery. Tumors could be either central or peripheral in location. Exclusion criteria for the study consisted of patients with small cell lung cancer and those treated for lung metastases. 

Patients were prescribed doses of 50-60 Gy in 3-5 fractions using either 3D-conformal technique or volumetric modulated arc therapy. No premedication with dexamethasone or other anti-inflammatory was used prophylactically during the course of treatment. Post-treatment surveillance imaging was obtained at three months, six months, and 12 months then every six months to three years, then annually. Disease recurrence was defined as post-treatment enlargement of the primary tumor on surveillance imaging with either FDG avidity on PET beyond that at diagnosis or biopsy. Since post-SBRT radiation fibrosis commonly occurs, enlargement of a mass-like consolidation on CT alone was not considered as recurrent disease. 

RRP was measured on surveillance CT using radiographic markers such as ground-glass opacities and airspace consolidation as a maximum dimension (long axis) and its perpendicular dimension. These measurements were used to calculate the maximal area of RRP using the calculation Area = pi * long axis * short axis.

RRP was evaluated and measured during a three-, six-, and twelve-month timeframe. Radiologic reports were also used to verify the presence of RP. Disease recurrence was the primary endpoint and was evaluated and categorized as local, regional, and distant failure. Disease recurrence was then analyzed relative to both presence and absence of RRP and the extent of RRP, as defined above, relative to the initial treatment size. 

Summary statistics are reported for clinical outcomes and characteristics. Fisher’s exact tests are used to assess bivariate associations where expected cell counts are < 5. The Kaplan-Meier method is used for survival estimation curves and a log-rank test used to assess whether these stratum-specific distributions differ significantly. Cox proportional hazards models are used to estimate the effects of RRP diagnosis on the hazard of disease progression. Univariable binary logistic regression models are used to estimate the unadjusted effects of individual RP measures on the odds of disease progression. 

## Results

Seventy-seven patients met inclusion and exclusion criteria from the database of patients treated with SBRT to be included in the study. The median follow-up was 24.3 months (IQR: 12.0 - 41.9). There were 72 patients (93.5%) that had RRP and five patients (6.5%) that did not. Patient and tumor characteristics are shown in Table 1. Of the patients in this cohort 59.7% are deceased, 28.6% had disease progression, and 66.2% had disease progression or death during the study period. Disease failure occurred in 28.6% of patients with 6.5% local only, 2.6% regional only, 7.8% distant only, and 11.7% with multiple recurrences. In total, there were 15.6% local, 9.1% regional, and 18.2% distant recurrences. The average maximum dimension of the tumor size upon presentation was 2.3 cm (SD 1.1 cm). The maximum dimension of the tumor consisted of only a single measurement of the tumor where it was largest. The average maximum dimensions of RRP at three, six, and 12 months were 3.5 cm (SD 2.2 cm), 5.0 cm (SD 2.1 cm), and 5.2 cm (SD 2.4 cm), respectively. The average areas of RRP at three, six, and 12 months were 6.9 cm², 10.9 cm², and 9.7 cm². The area of RRP was calculated using two dimensions of the tumor. A majority (81.8%) of patients who had RRP were grade 1 (radiographic only) whereas symptomatic grade 2 RP was present in 11.7% (Table 1). The majority of patients with symptomatic RP were treated with oral prednisone. 

**Table 1:** Clinical Characteristics of a Sample of Non-Small Cell Lung Cancer Patients Treated with Stereotactic Body Radiation Therapy (SBRT) LLL: Left Lower Lobe, LUL: left upper lobe, RML: Right middle lobe, RUL: Right upper lobe, RLL: Right lower lobe, SD: Standard deviation, IQR: Interquartile range

Variable	n	Summary Statistic
Tumor Location, n (%)	77	
Missing		3 (3.90)
Lingula		1 (1.30)
LLL		8 (10.39)
LUL		26 (33.77)
RLL		12 (15.58)
RML		3 (3.90)
RUL		24 (31.17)
Lung Toxicity, n (%)	77	
None		5 (6.49)
Grade 1 Toxicity		63 (81.82)
Grade 2 Toxicity (Symptomatic)		9 (11.69)
Pneumonitis, n (%)	77	
Yes		72 (93.51)
No		5 (6.49)
Local Failure, n (%)	77	
Yes		12 (15.58)
No		65 (84.42)
Regional Failure, n (%)	77	
Yes		7 (9.09)
No		70 (90.91)
Distant Failure, n (%)	77	
Yes		14 (18.18)
No		63 (81.82)
Death, n (%)	77	
Missing		6 (7.79)
Yes		46 (59.74)
No		25 (32.47)
Disease Progression, n (%)	77	
Yes		22 (28.57)
No		55 (71.43)
Number of Failures, n (%)	77	
0		55 (71.43)
1		13 (16.88)
2		7 (9.09)
3		2 (2.60)
Type of Failure(s), n (%)	77	
Multiple Failures		9 (11.69)
Distant Failure		6 (7.79)
Regional Failure		2 (2.60)
Local Failure		5 (6.49)
No Failure		55 (71.43)
Maximum Tumor Dimension, Mean (SD)	74	2.29 (1.06)
Secondary Tumor Dimension, Mean (SD)	72	1.79 (0.84)
Maximum Pneumonitis Measure, 3 Months, Mean (SD)	68	3.25 (2.27)
Minimum Pneumonitis Measure, 3 Months, Mean (SD)	68	2.06 (1.57)
Maximum Pneumonitis Measure, 6 Months, Mean (SD)	63	4.70 (2.34)
Minimum Pneumonitis Measure, 6 Months, Mean (SD)	63	2.87 (1.51)
Maximum Pneumonitis Measure, 12 Months, Mean (SD)	51	4.82 (2.68)
Minimum Pneumonitis Measure, 12 Months, Mean (SD)	51	2.87 (1.69)
Radiation Pneumonitis Area, 3 Months, Mean (SD)	77	6.43 (8.56)
Radiation Pneumonitis Area, 6 Months, Mean (SD)	77	10.22 (9.76)
Radiation Pneumonitis Area, 12 Months, Mean (SD)	77	9.08 (12.99)
Pneumonitis Area/Tumor Area, 3 Months, Mean (SD)	77	2.18 (3.39)
Pneumonitis Area/Tumor Area, 6 Months, Mean (SD)	77	4.02 (5.43)
Pneumonitis Area/Tumor Area, 12 Months, Mean (SD)	77	3.43 (4.71)
Follow-up Time (months), Median (IQR)	72	24.33 (12.02 – 41.89)

**Figure 1:**
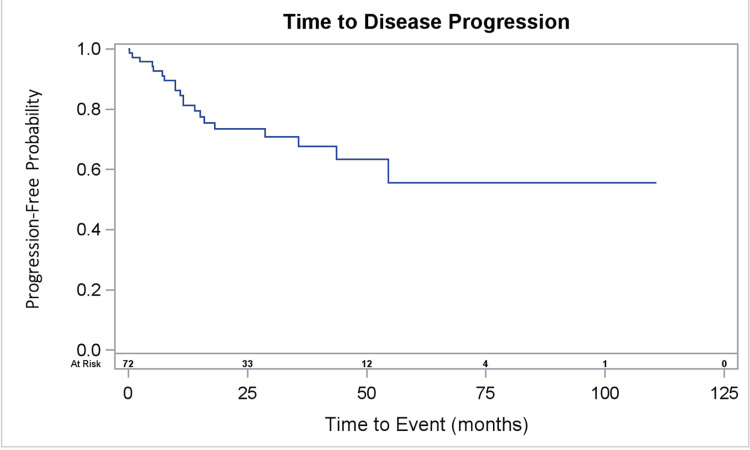
Overall Time to Disease Progression of a Sample of Non-Small Cell Lung Cancer Patients Treated with Stereotactic Body Radiation Therapy (SBRT)

**Figure 2:**
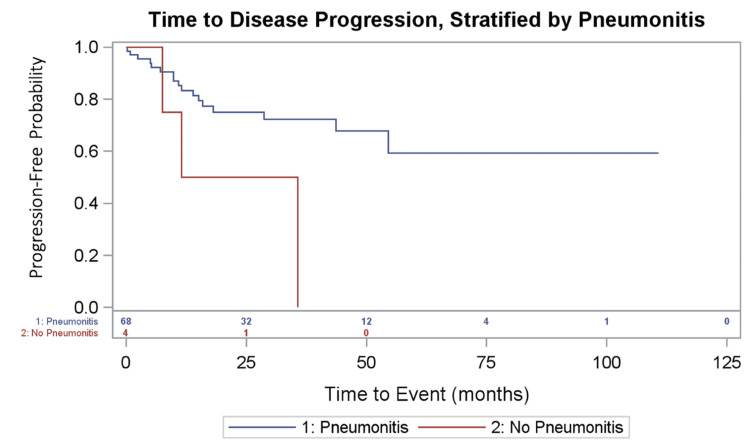
: Progression-Free Survival, Stratified by Radiation Pneumonitis

Disease progression occurred in 25.0% of patients with RRP and in 80.0% of patients without RRP, p=0.02, as shown in Table 2. Median time to disease recurrence or progression was unobserved for patients with RRP (28.7 - unobserved), and was 35.7 months (11.5 - 35.7) for patients without RRP (p = 0.04). Figure 1 and Figure 2 depict the time to disease progression.

**Table 2:** Association Between Radiation Pneumonitis & Disease Progression

	Disease Progression		p
Radiation Pneumonitis	Yes	No	
Yes (%)	18 (25.0)	54 (75.0)	0.02*†
No (%)	4 (80.0)	1 (20.0)	

Table 3 shows the distribution of failure type for the patient population. On univariate analysis, the hazard of disease progression was lower for patients with RP than it was for patients without RP (HR 0.29; 95% CI [0.08, 1.00], p=0.05) (Table 4). On multivariate analysis, presence of RP decreased the hazard of disease progression (HR 0.20; 95% CI [0.05, 0.76], p=0.02) when controlling for presence of tumor size as shown in Table 4. 

**Table 3:** Association Between Radiation Pneumonitis & Failure Type

	Failure Type				
Radiation Pneumonitis	Multiple Failures	Distant Failure	Regional Failure	Local Failure	No Failure
Yes (%)	8 (11.1)	5 (6.9)	2 (2.8)	3 (4.2)	54 (75.0)
No (%)	1 (20.0)	1 (20.0)	0 (0.0)	2 (40.0)	1 (20.0)

**Table 4:** Effects of Radiation Pneumonitis on the Hazard of Disease Progression REF: Reference category

Predictor	Effect	Unadjusted HR (95% CI)	p	Adjusted HR (95% CI)	p
Radiation Pneumonitis	Yes vs. No (REF)	0.29 (0.08, 1.00)	0.05	0.20 (0.05, 0.76)	0.02*
Maximum Tumor Dimension	1-unit increase	-	-	1.43 (0.91, 2.24)	0.13

When evaluating the area of RRP, none of the three RP measurements (maximum RP dimension, RP area, and RP area:tumor area ratio) demonstrated a significant effect on disease progression at the three-, six-, or 12-month time points as shown in Table 5. 

**Table 5:** Unadjusted Effects of Radiation Pneumonitis Measures on the Odds of Disease Progression

Variable	Time	n	OR (95% CI)†	p
Maximum Pneumonitis Measure	3 months	68	0.95 (0.76, 1.19)	0.68
	6 months	63	0.97 (0.77, 1.21)	0.76
	12 months	51	0.90 (0.71, 1.14)	0.38
Radiation Pneumonitis Area	3 months	77	1.02 (0.97, 1.08)	0.39
	6 months	77	1.01 (0.97, 1.07)	0.57
	12 months	77	0.99 (0.96, 1.03)	0.71
Radiation Pneumonitis Area / Tumor Area	3 months	77	1.01 (0.88, 1.16)	0.91
	6 months	77	1.02 (0.93, 1.11)	0.72
	12 months	77	1.01 (0.91, 1.12)	0.87

## Discussion

In this study we aimed to examine the role of RP in patients who received SBRT in relation to the progression of their disease. We found that most patients who received SBRT had RRP, and that it appeared to be protective with decreased risk of cancer recurrence. In our cohort, patterns of failure are in line with other published series with distant recurrence as a leading site of failure. Importantly, we found that progression-free survival was significantly improved among patients who developed RRP compared to those who did not. 

Several studies demonstrated that symptomatic RP occurs after SBRT with relatively low rates of about 10% [10,20,21]. However, the presence of asymptomatic RRP is very common in clinical practice. Our hypothesis was that the presence of RRP following SBRT may serve as an immunological protective mechanism against local tumor recurrence and further disease progression. There is a large body of evidence suggesting enhancement of the immune system after radiation beginning with the promotion of tumor antigens stimulating the immune system and enhancement of T cell recognition and killing [22,23]. SBRT may result in tumor cell kill through multiple mechanisms including obliteration of tumor vasculature, alteration of the tumor microenvironment, antigen release, and a local inflammatory response in addition to direct and indirect DNA damage. CD8+ T cells have been shown to be important in this local inflammatory process [18]. Hallahan et al. found that there is an increase in expression of cellular adhesion molecules in response to radiation, and these changes in the vascular endothelium lead to increase extravasation of the immune cells [19]. This can help explain our findings of why patients with identifiable RRP have lower rates of disease progression. 

Patients with early-stage lung cancer treated with SBRT still have high rates of distant failure ranging from 16-33% [2,24-27]. Strategies to decrease the risk of distant failure are an important area to improve upon with further research. Several ongoing studies are looking to initiate a greater systemic immune response, hopefully aided by the local SBRT, by combining checkpoint inhibiting agents sequentially and/or concurrently with SBRT. The PACIFIC-4 study is investigating the effects of 24 months of durvalumab (anti-PD-L1 monoclonal antibody) compared to placebo in patients with early-stage NSCLC treated with SBRT. The primary outcome of this study is progression-free survival (NCT03833154) [28]. In addition, a SWOG/NRG Oncology study is investigating the addition of six months of atezolizumab (anti-PD-L1 monoclonal antibody) to SBRT in early-stage NSCLC with the primary outcome of overall survival (NCT04214262) [29]. 

To our knowledge there are no studies prospectively investigating the impact that RRP may have on progression-free survival or overall survival in patients with RRP compared to those without RRP. Our study found similar rates of asymptomatic and symptomatic RP as previous studies. In addition, there appeared to be a difference in PFS in patients with RP compared to those without any evidence of RP on imaging. 

This study has some limitations including a small sample size randomly taken from a larger group of dataset for this retrospective analysis. The limited number of patients may have skewed toward the majority showing RRP, when this number may be lower. In addition, we evaluated for RRP using area rather than volume leading to some additional limitation in the true extent of the inflammation. If similar work is to be done on a larger scale, then propensity matching of patients with and without RP would be considered to validate this hypothesis. Additionally, there are potentially radiomic characteristics of RRP that may further risk stratify these patients where variations or evolution of the post-treatment radiographic characteristics may be additionally predictive of outcome. Given that this study is retrospective, peripheral blood for testing of circulating activated CD8+ T-cells was not performed though would be interesting in future investigation. 

Further work will be done to explore extent of RRP on disease recurrence and mortality. Future analyses will be needed to validate this data with a larger dataset, to externally validate the information at other institutions, and eventually perform prospective evaluations of these endpoints. 

## Conclusions

Most patients with NSCLC treated with SBRT developed RRP. Patients with RRP had significant improvement in progression-free survival compared to those patients who did not develop RRP. This local inflammatory response may act as an immunological protective method to decrease the risk of cancer recurrence. Further analysis is needed with a larger data set to evaluate these endpoints. 
